# Investigating the Effects of Differential Learning on Golfers’ Pitching Performance as a Function of Handicap

**DOI:** 10.3390/ijerph191912550

**Published:** 2022-10-01

**Authors:** Miguel Valença, Diogo Coutinho, Wolfgang Schöllhorn, Nélson Ribeiro, Sara Santos

**Affiliations:** 1 University of Maia, UMAIA, 4475-690 Maia, Portugal; 2 Department of Sports Sciences, Exercise and Health, University of Trás-os-Montes and Alto Douro, 5000-801 Vila Real, Portugal; 3 Research Center in Sports Sciences, Health Sciences and Human Development, CIDESD, CreativeLab Research Community, 5000-801 Vila Real, Portugal; 4 Department of Training and Movement Science, Institute of Sport Science, Johannes Gutenberg-University Mainz, 55099 Mainz, Germany; 5 Faculdade de Motricidade Humana, Universidade de Lisboa, 1495-751 Lisboa, Portugal

**Keywords:** differential learning, handicap, movement variability, club speed, carry distance, TrackMan 4

## Abstract

Traditionally, golf instruction has been oriented toward imitation of role models, guided by errors that surround a channel of supposedly correct repetition. Recent motor learning approaches relying on the dynamics of living systems suggest the inclusion of additional noise during practice for supporting players’ movement exploration and improving adaptability that in consequence will lead to increased performance. While the effectiveness of this approach has now been demonstrated in many sports, research exploring the effects of differential learning (DL) in golf is scarce, especially when considering different shot distances and players with various handicap levels. Therefore, the purpose of this study was to compare the effects of an enriched learning and information intervention as opposed to a more constrained approach, on the pitching performance of golfers with different handicaps from different distances. A total of 29 adolescent golfers with an average experience of 7.8 years were divided into a DL (*n* = 15) and a repetitive-oriented (RB, *n* = 14) group. Both groups were further compared dependent on their handicap level (DL, low handicap *n* = 7, high handicap *n* = 8; RB, low handicap *n* = 5, high handicap *n* = 9). The TrackMan 4 was used to measure the shot performance for 20 m, 35 m, and 50 m distances (10 shots from each distance) based on a pre- and post-test design. Each group performed the same number of trials (*n* = 270, 9 executions per distance per session) across 10 sessions. Analysis of covariance (ANCOVA) was used for the statistical analysis, using the pre-test as covariate and the post-test as dependent variable. The DL group revealed advantageous adaptations in the attack and face angle (*p* ≤ 0.05), while also in the dynamic loft (*p* ≤ 0.05), mostly for the 35 m and 50 m. In addition, this intervention led to improvements in the score, club head speed, and carry distance for the 50 m when compared to the RB (*p* ≤ 0.05; small effects). The low handicap players from the DL group also revealed adaptation in the angles’ variables (*p* ≤ 0.05) when compared with high handicap players, who improved the score (*p* ≤ 0.05) in all distances after intervention. The low handicap players from the RB group improved the score (*p* ≤ 0.05) and club speed (*p* ≤ 0.05) for the 20 and 35 m, while the high handicap golfers revealed higher improvements for these variables only in the 50 m distance condition. Overall, coaches may incorporate approaches into their skill training that increase the number of opportunities to improve the performance of both experienced and non-experienced players by promoting the adaptability of movement patterns.

## 1. Introduction

Golf is a popular sport performed in more than 130 countries. This sport consists of a long duration activity, usually lasting from 3.5 to 6 h for a round of 18 holes [1]. Thereby, a golfer’s success is dependent on the distance and lateral deviation of each shot [2,3]. The main aim is to use the lowest possible number of strokes to hit all the holes of the golf course, leading to a lower handicap score [4]. The clubhead speed is a critical determinant for achieving higher carry distances when considering long-distance shots [5,6]. Golfers with a lower handicap (i.e., better performance levels) showed higher speed values of club head speed than golfers with higher handicap [7]. This difference may be related to the fact that lower handicap golfers are better able to activate muscles sequentially, whereas novice golfers are more likely to perform rigid movements [8], resulting in a lower ability to rotate the trunk and transfer energy to the ball than more experienced players [3]. It seems that more advanced golfers adjust the level of movement variability during the different swing phases, as they increase the variability during the downswing phase, while decreasing it during the impact point [2]. Different visual search strategies [9], a better perception of distance by the golfers with lower handicap [2], while also the higher control of clubhead impact [10] may also contribute to such differences in performance [2]. An additional determining factor that distinguishes better and less skilled golfers is their ability to adapt their movement pattern during the shot [2].

For improving the efficiency of a golf swing, coaches have developed various strategies that may help to increase the energy transfer during the swing to ensure a proper club head speed [11,12]. Most of these training approaches are grounded in the improvement of physical capabilities [5,13,14]. Alternatively, considering kinematic or technical development as the main prerequisite for activating each muscle in an appropriate sequence during a golf stroke, different training strategies have been proposed [12,15,16]. Traditional approaches in motor performance emphasized the use of prescriptive instructions and feedback during repetitive training, thereby constraining and guiding the athlete’s movement towards a movement prototype [17]. Latently, this approach tends to focus on increased movement consistency [13]. In contrast, more current approaches have emphasized the importance of group specific movement techniques as it may rely on features and characteristics of several athletes [15,16,18], which has contributed to a shift in the research scope towards more focus on individual training [19,20,21]. In addition, adding variability during training practice enhances learning and performance in fine and in gross motor movements [22,23]. Based on these considerations, some studies have explored how adopting tasks grounded in additional variability may enhance golfers’ performance. A single-session pre- and post-test design was used to examine the effect of different levels (i.e., low, moderate, and high) of contextual interference (CI) on the learning of golf putting and pitching skills, without allowing errors, in male undergraduates [16]. It was found that the group under high CI improved both skills more than the moderate and low CI training groups [16]. This study provided evidence that players seem to enhance their performance when facing acute training interventions based on tasks by interleaving the repetitions with other movements. However, further research exploring training interventions grounded in variability are required, as the players’ acute and long-term effects are not always consistent.

The benefits of practicing with variability combined with the stronger emphasis on the individuals’ techniques have become a focus of attention in recent research [24,25]. In this respect, the differential learning (DL) approach has been used to add variability in the form of additional movement noise during players’ training routines. The DL approach focuses on increasing the observed fluctuations in the players’ movement patterns by neither repeating a movement nor providing augmented feedback during or following the task [26,27,28,29], to foster adaptive mechanisms in the action and apperception system of the players [24,30]. This approach suggests that the addition of stochastic perturbations will enhance individual and self-organized learning where no explicit information about a possible solution is provided [28,29]. For that purpose, players are challenged to perform unusual and also non-representative movements that not only inspire them to refine the existent motor patterns [26,30] but also lead to brain states that are more advantageous for learning processes once applied in a random sequence [31,32]. Athletes could be instructed to perform or even create their own movements, such as playing football with both hands on the hips or even finding by themselves several variants of the arms in front of the body to also stimulate the whole spectrum of trunk muscles by producing the corresponding levers [24]. By being exposed to a wider range of movements, DL allows the players not only to develop momentary individual solutions but even more to prepare athletes to stay individually adaptive to constantly changing internal and external boundary conditions in the future [28,33]. Under this perspective, DL tenets sustain that the fluctuations added during the practice would help each individual to explore and refine their optimal and situational movement patterns [28,29]. Analogous to the training of artificial neural nets in DL, the human neuronal system is trained in a noisy manner to increase the performance afterwards [34].

Based on these principles, some studies have examined the impact of long-term DL-supported training on the performance of soccer players compared to a more constrained traditional group (i.e., repetitive-based practice -RB) [30,35]. After a 5-month intervention, the under-13 and under-15 players who engaged in DL revealed a more regular behavior, which may indicate higher game knowledge, while also improving their in-game technical and creative performance [30]. Moreover, positive effects were found after a 10-week intervention under the DL principles, in which under-15 and under-17 soccer attackers improved their technical and creative behavior, while their positioning become more unpredictable [36]. In addition, higher rates of improvement were found in both studies for the younger aged groups, possibly because of previously having been less exposed to repetitive and structured practice [30,35]. These advantageous effects of DL seem to be amplified by increasing the duration of DL intervention. An intervention study on intensive goal kicking in football over three months with juvenile females [37] led to even higher learning rates than in earlier studies for the duration of 6 weeks [27]. Besides numerous corroborating studies in different sports [38,39], two studies on golf pointed to the same direction. One study explored how a golf DL intervention affected the performance of golfers who were grouped into an RB group and DL group. The intervention was implemented over a three-month period. The results showed that DL outperformed the traditional intervention, mainly in the score, during a golf course consisting of nine holes [40]. Similar findings resulted from another golf study [36]. However, a possible dependence of the training approaches followed on the level of performance or age has yet to be established.

Earlier studies exploring the DL effects in football players showed distinct effects when considering the age of participants [30,35]. However, when considering the players’ experience, different results may occur. Accordingly, it is expected that golfers with a low handicap score show higher variability between shots compared with players with higher handicap score [41]. Thus, it may be possible that the fluctuations resulting from variations in geometry (e.g., varying the ball type), in velocity (e.g., playing under visual occlusion), or even in the rhythm (e.g., adjusting the body starting position in relation to the ball) may limit or emphasize the individual variability, thereby affecting players’ performance [28,34,41]. Therefore, the aim of this study was to explore the effect of a training intervention based on DL on the pitching performance and how these effects depend on a golfer’s expertise level (low and high handicap scores).

## 2. Materials and Methods

### 2.1. Participants

Participants were 29 adolescent Portuguese golfers (23 male and 6 female; age: 16.03 ± 2.47 years, body mass: 64.62 ± 9.86; height: 172.07 ± 9.86 cm, handicap level: 11.40 ± 12.01, playing experience: 7.38 ± 3.42 years) belonging to the same golf club. All golfers had at least 1 year of experience in golf. All participants were grouped into the DL group (i.e., differential learning group) and the control group (i.e., repetitive-based group) as well as into low and high handicap groups. The division into the players’ expertise level followed the local National Golf Federation, stating that expert players present a handicap score equal to or lower than 5.0. Table 1 summarizes the golfers’ characteristics according to the DL or RB and low handicap and high handicap profile. A written and informed consent was provided to the coaches, players, and by their legal guardians, as well as by the club, before the beginning of the study. All golfers were notified that they could withdraw from the study at any time. The study protocol followed the guidelines and was approved by the local Ethics Committee of the Research Center in Sports Sciences, Health Sciences and Human Development (UIDB/4045/2020) and conformed to the recommendations of the Declaration of Helsinki.

### 2.2. Training Intervention

After allocating the golfers into the DL group and RB group, both engaged in the same amount of practice. The training program consisted, for all groups, of performing 27 pitching executions per session (*n* = 9 pitching executions per each distance—20 m, 35 m, and 50 m—see Table 2), ending up with a total of 270 shots in 10 sessions.

For the RB group, a constant practice approach was adopted, that is, the golfers performed the 9 repetitions using the same technique and the same distance before moving forwards to the following distance. The RB group received augmented feedback after each shot. Before each shot, the coach provided prescriptive instructions on how to adjust the movement technique focused on the distance, direction, and contact between the iron and the ball.

**Figure 1:**
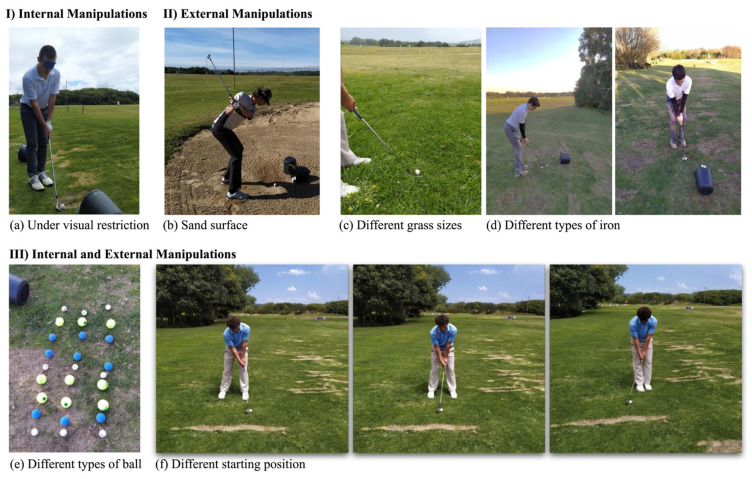
Examples of the fluctuations (**a**–**f**) enacted to the DL group. Note: (**a**) internal manipulations by performing the golf shot under visual restriction using an eye patch; external manipulations by performing the golf shot in (**b**) different surfaces—sand; (**c**) different grass sizes; (**d**) different types of iron; internal and external manipulations by (**e**) varying the types of ball used; (**f**) different starting positions.

The DL group performed the same number of pitching executions in total and per session, however, under the DL principles (Figure 1 and Table 2). Every shot was performed in a different way accordingly to internal manipulations (i.e., visual restriction), external (i.e., type of surface, such as playing on sand or in a pitch with high size grass, type of iron in size and loft, different types of balls), or both (i.e., distances, starting distance to the ball, or varying the ball position relative to the golfer’s feet). Thereby, the athletes performed every shot in a different way, meaning that the first shot was performed to the 20 m, the second to the 35 m, and the last to the 50 m. In the DL group, and considering that each shot was unique, players were only instructed regarding how to perform the momentary shot (e.g., referring that the body position should be adjusted to have the ball aligned with the right foot) [24,30,35]. The conditions or instructions for the repetitive (control) practice and the DL practice group, are outlined in Table 2. Both groups were further divided into low and high handicap groups.

### 2.3. Testing Session and Measurements

The radar system TrackMan 4 (TrackMan A/S, Vedbæk, Denmark) was used to measure the golfer’s performance for the following variables: score, club speed, carry distance, side, attack angle, face angle, and dynamic loft. The score is a value between 0 and 100 and is determined by the distance of the ball (i.e., the carry) and the direction to the destination [42]; the club speed refers to the speed of clubhead at the impact point; the carry distance reports the distance covered in meter by the ball aerial trajectory; the side measures the distance at the time that the ball first hit the ground; the attack angle refers to vertical movement of the club during the impact; the face angle consists of the orientation of the club face in relation to the target line during the impact; and the dynamic loft measures the orientation of the club face in relation to the plumb line, at the impact [10]. The TrackMan 4 is used by low and high handicap golfers based on the ability to provide accurate, reliable, and instantaneous feedback [42]. This device measures and displays the trajectory of every shot from different distances. The monitor was prepared according to manufacturer’s requirements, that consists of positioning it 2.8 m behind the impact area, while the screen being positioned 5 m in front of this area. For the testing sessions, the golfers were instructed to use their own wedges, while the same ball was used for all the golfers. No familiarization session was performed before the testing sessions as the golfers were used to the practice task and the measurement instruments during both pre- and post-test situations/conditions. Before the testing session, the players engaged in a 10-min warm-up consisting of mobility and dynamic stretching exercises. They then performed an additional 5-min technical warm-up consisting of hitting 3 balls from each measuring distance. The pre- and post-test consisted of performing 30 shots from 3 different distances: (a) 20 m; (b) 35 m; and (c) 50 m. For each distance, the golfers performed 10 trials. The tests were performed in a blocked order, since the golfers always performed 10 trials from a 20 m distance, followed by 10 trials from a 35 m distance, and then the last 10 trials from a 50 m distance. Subjects were instructed to hit all golf balls using their swing technique as they would use during competition. All the tests were conducted in a natural turf pitch. The ball was hit to a target (i.e., regular golf flag) at a 20 m, 35 m, or 50 m distance. Between each distance, there was a 1-min resting time to ensure proper calibration of the measurement instruments, and in general, each player’s testing session lasted for 15 min. All measurements for each player were performed at the same time of the day to minimize possible effects of the circadian rhythm.

### 2.4. Statistical Analysis

The sample size was calculated with G∗Power (Version 3.1.5.1, Institut für Experimentelle Psychologie, Düsseldorf, Germany) for an effect size of 1.0, an α of 0.05, and a power of 0.8 [43,44]. The total sample size computed by this was 24, consisting of 12 participants per group. Individual and mean changes from pre- to post-test for the considered group activities were graphically represented and the variation from each test moment was expressed in percentages (mean ± SD) [45]. All data were assessed for outliers and assumptions of normality. Because of nonequality in the pre-test, analysis of covariance (ANCOVA) was used for analysis, with post-test values as the dependent variable and pre-test values as the covariate. For each ANCOVA, partial eta-squared (η2) was calculated. Values of 0.01, 0.06, and above 0.14 were reported as small, medium, and large, respectively [46]. Statistical significance was set at *p* ≤ 0.05 and calculations were carried out using SPSS software V26.0 (IBM SPSS Statistics for Windows, Armonk, NY, USA, IBM Corp.). Complementary, a specific spreadsheet for pre–post crossover trials, was applied to document the magnitude effects between protocol interventions on players’ performance measures. These effects were estimated in percent units based on log-transformation and 95% confidence limits were used to express the uncertainty in the estimation. Additionally, standardized (Cohen) mean differences with the corresponding 95% confidence intervals were computed as the magnitude of observed effects, using the following thresholds: <0.2, trivial; <0.6, small; <1.2, moderate; <2.0, large; and >2.0, very large [47].

## 3. Results

### 3.1. Comparing the DL (n = 15) and the RB Group (n = 14)

**Figure 2:**
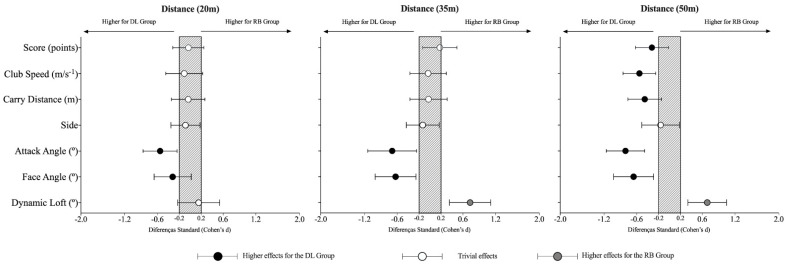
Standardized (Cohen) differences in golf shot performance for the different variables according to the distance and training intervention. Error bars indicate uncertainty in the true mean changes with 95% confidence intervals. DL = differential learning; RB = repetitive-based group; M = mean; SD = standard deviation.

Table 3 and Figure 2 present the descriptive and inferential effects between the RB and DL group according to the three distances. Although statistically significant effects were identified in all distances, larger differences were identified for the longer distances, mainly for the 50 m distance. Albeit increasing values, the DL group showed statistically significant lower values in the side values compared to the RB group for the 20 m distance (F = 4.71, *p* < 0.05, trivial effects). In addition, a higher negative value for the attack angle (F = 14.1, *p* < 0.001, small effects) was identified.

For the 35 m distance, statistically significant differences between both groups were identified in the attack angle (F = 4.62, *p* = 0.033, moderate effects), the face angle (F = 8.34, *p* = 0.004, moderate effects), and the dynamic loft (F = 11.66, *p* < 0.001, moderate effects). In this respect, higher negative attack angle values were found for the DL group, while an increase in face angle and a decrease in the dynamic loft compared to the RB group could be observed.

When comparing the shot performances for the 50 m distance between the DL and RB groups, results showed statistically significant effects in all variables from the side. After the training intervention, the DL group revealed higher values for the score (F = 12.13, *p* < 0.001, small effects), club speed (F = 9.02, *p* = 0.003, small effects), and the carry distance (F = 9.68, *p* = 0.002, small effects) compared to the RB group. In addition, the DL group presented higher values of the face angle (F = 8.88, *p* = 0.003, moderate effects), higher negative values for the attack angle (F = 19.8, *p* < 0.001, moderate effects), while presenting lower values for the dynamic loft (F = 13.88, *p* < 0.001, moderate effects) in comparison to the RB group.

### 3.2. Differences within the Golfers of the DL Group between Low Handicap (n = 7) and High Handicap (n = 8) Scores

**Figure 3:**
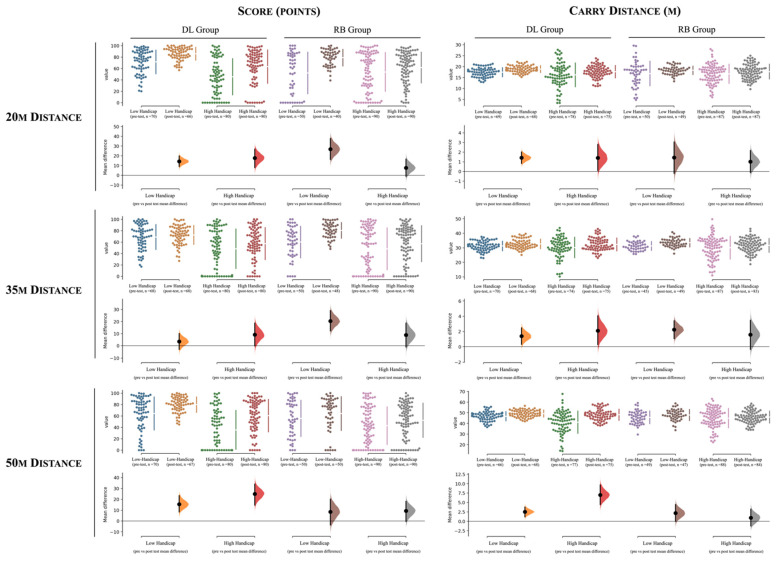
The mean difference for 4 comparisons according to: (i) the score and carry distance and to (ii) distances (20 m, 35 m, and 50 m) are shown in the above Cumming estimation plot. The raw data are plotted on the upper axes; each mean difference is plotted on the lower axes as a bootstrap sampling distribution. Mean differences are depicted as dots; 95% confidence intervals are indicated by the ends of the vertical error bars [45]. DL = differential learning; RB = repetitive-based.

**Figure 4:**
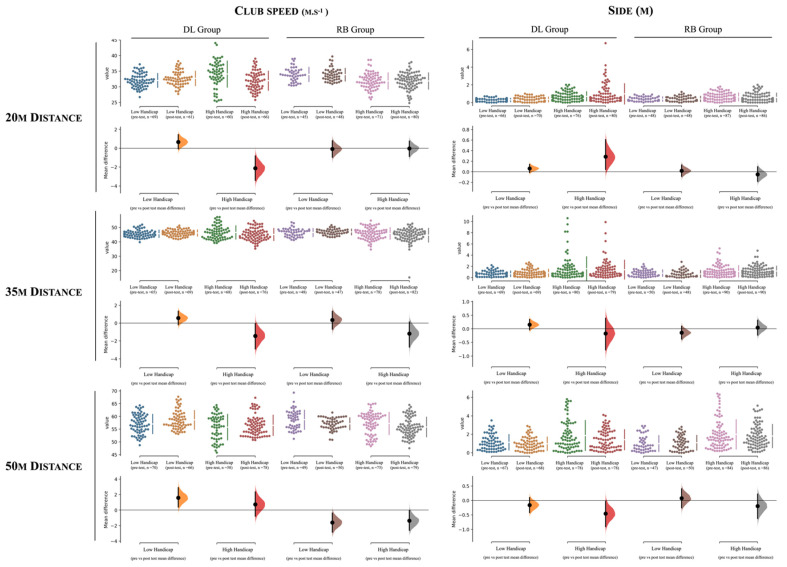
The mean difference for 4 comparisons according to: (i) the club speed and side and to (ii) distances (20, 35 m, and 50 m) are shown in the above Cumming estimation plot. The raw data are plotted on the upper axes; each mean difference is plotted on the lower axes as a bootstrap sampling distribution. Mean differences are depicted as dots; 95% confidence intervals are indicated by the ends of the vertical error bars [45]. When the mean difference is negative, should the distribution curve show on the other side? In the present way, it is somewhat confusing. DL = differential learning; RB = repetitive-based.

**Figure 5:**
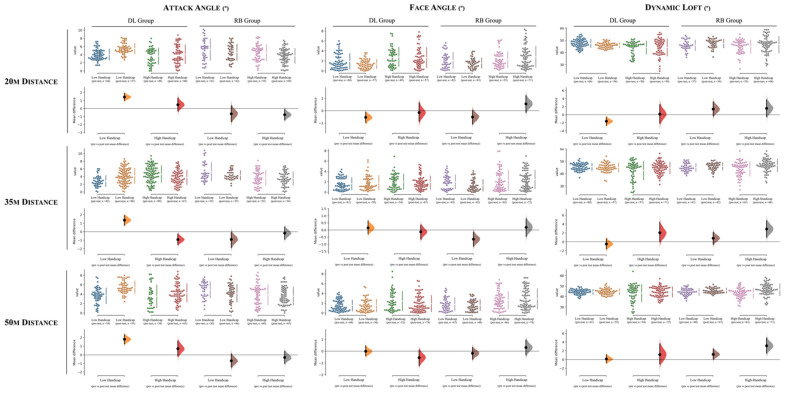
The Cumming estimation plot of the mean difference for 4 comparisons according to: (i) the attack angle, face angle and dynamic loft; and to (ii) distances (20 m, 35 m and 50 m). The raw data is plotted on the upper axes; each mean difference is plotted on the lower axes as a bootstrap sampling distribution. Mean differences are depicted as dots; 95% confidence intervals are indicated by the ends of the vertical error bars [45]. DL = differential learning; RB = repetitive-based.

Table 4 and Figure 3, Figure 4 and Figure 5 contain the descriptive and inferential effects between the low and high handicap scoring groups for the DL intervention (i.e., experimental group). Descriptive results between comparisons can also be found in Figure 3, Figure 4 and Figure 5 (*Cumming estimation plots*). For interpretation purposes, each dot represents a player’s pitching shot attempt. In all figures, the left side of each variable represents the values from the DL group, while in the right side corresponds to the RB group. For each group, the differences between players’ experience are presented (e.g., a mean difference of 18% in players’ score points were found between the pre- to post-test assessment for the high handicap players, whereas this value decreases to ~10% in the pre- to post-test comparison of the low handicap group).

For the 20 m distance, the high handicap score group revealed higher improvements in the score (F = 10.1, *p* = 0.002, small effects) while lower negative values for the attack angle (F = 4.59, *p* = 0.035; trivial effects) compared to the low handicap score group.

The training intervention for the 35 and 50 m distance showed similar trends between the groups. Accordingly, higher improvements in scores (35 m, F = 7.61, *p* = 0.007; 50 m, F = 13.81, *p* < 0.001, small effects) were found for the high handicap group (i.e., low level). In contrast, higher improvements for club speed (35 m, F = 4.56, *p* = 0.035; 50 m, F = 22.1, *p* < 0.001, moderate and small effects for the 35 and 50 m distance respectively) were identified for the low handicap group. In addition, an increase in the negative attack angle (35 m, F = 4.17, *p* = 0.045; 50 m, F= 3.96, *p* = 0.05, moderate and small effects for the 35 and 50 m distance respectively) and a decrease in dynamic loft (35 m, F = 11.3, *p* = 0.001; 50 m, F = 10.36, *p* = 0.002, moderate effects) for the low handicap group were found, while an increase in the positive face angle (35 m, F = 8.04, *p* = 0.005; 50 m, F = 5.71, *p* = 0.018, moderate effects) values in comparison to the high handicap group were found.

### 3.3. Differences within the RB Group between Low Handicap (n = 5) and High Handicap (n = 9) Golfers

Table 5, and Figure 3, Figure 4 and Figure 5 present the descriptive and inferential effects between the low handicap and high handicap groups for the RB approach (i.e., repetitive-based group).

The results show statistically significant differences in the score and club speed in all distances and in the carry distance for the 35 m distance. In this respect, higher improvements following the intervention for the score (20 m, F = 15.59, *p* < 0.001, small effects; 35 m, F = 17.12, *p* < 0.001, small effects) and club speed (20 m, F = 19.01, *p* < 0.001, small effects; 35 m, F = 9.56, *p* = 0.002, small effects) were found for the low handicap group when considering the 20 and 35 m distances, while in contrast, these were found for the high handicap group when considering the 50 m distance (score, F = 3.92, *p* = 0.05, trivial effects; club speed, F = 6.47, *p* = 0.012, trivial effects). In addition, the low handicap group also improved the carry distance more for the 35 m distance in comparison to the high handicap group (F = 8.34, *p* = 0.005, small effects).

## 4. Discussion

This study aimed to explore the effects of a DL on the pitching performance in golf. Additionally, this study aimed to analyze how these effects depend on the golfers’ experience (golfers with low and high handicap score). In general, the training intervention induced a higher impact for the long distance (i.e., 50 m) as the results were statistically significant and with higher effects for almost all variables in this area.

### 4.1. Differences of the Intervention between the DL and RB Training Group

Most intervention-based studies on golf technique have been focused on non-specific training tasks, mainly by exploring the relation of improving specific physical abilities on the shot performance. Examples of these interventions are training with weights or exposing the players to plyometric and flexibility interventions [4,5,14,48,49]. The major feature from this study was the inclusion of golf-specific exercises to improve golfers’ performance similar to Blumhoff and Vernekohl [36] and Wewetzer [40], however, to understand its effects on pitching performance and performance level in more detail. In addition, classical motor learning theories have argued for the importance of repeating the technique to be learned to enhance the cognitive and motor control [11,12]. In this regard, for many years movement in golf practice variability was considered unnecessary or even detrimental for fear of acquiring wrong movement patterns. However, recent reports have shed a different light on its importance to improve players’ learning progress while also developing their movement adaptability [17,21,22,30]. In addition, golfers seem to be able to face time related variability without compromising short outcome performance [15]. The results from this study provide evidence for an adaptation in the player’s movement technique after the content related variable DL interventions. For the 20 m distance (see Table 3), the main differences between the control and DL group were related to the side and attack angle of the ball. The first parameter reports how lateral movement related to the straight target line leads to the ball deviating, while the second refers to changes in the vertical movement of the golf club when hitting the ball. Thereby, higher negative values indicate a path closer to the ground and positive angles indicate a higher trajectory in the beginning of the flight [50]. Regarding the side variable, the DL group outperformed the RB group, as it presented values close to 0. For this parameter, values close to 0 mean that there is less lateral deviation from the intended target. In addition, despite not being statistically significant, there was a slightly bigger effect on the face angle favoring the DL group. The face angle refers to the angle of the club face in relation to the target line, whereas negative values mean that the club face is pointed to the left of the target, while positive ones mean that the club face is pointed to the right of the target (i.e., for right-handed golfers). In this study, the RB group face angle values increased (from −0.89° in the pre-test to −1.16° in the post-test), which may justify the increase in the side parameter. In fact, it has been shown that both the face angle and attack angle seem to affect the side parameter [50].

The angular conditions of club head and ball flight (i.e., attack angle, face angle, and dynamic loft) were those that were most stressed by the intervention across the different distances. According to the DL theory, players were exposed to a wide range of variations (e.g., different foot positions, different posture, with visual occlusion, playing on different surfaces, with different starting ball positions,), which may amplify their adaptability and movement patterns [26,28,30,33]. Golfers were systematically confronted with new and non-representative movement patterns, which required them to adapt different phases of the swing, and in consequence, the way in how they hit the ball. In this respect, the fluctuations that were created according to DL theory may not have only allowed the golfers to explore a wider range of different golf shot technique variations [12,23], by amplifying their opportunities for action, but also may have led to advantageous brain states for learning [31,32]. These results may support early [51,52] and recent statements reporting the importance of movement variability to enhance performance [14,15,17,18]. When considering the changes in ball trajectory, a trend was found towards negative angles for the attack angle by the DL group. It seems that golfers with a lower handicap prefer to adjust the attack angle according to the environmental requirements and usually adopt a ball trajectory close to the ground [53]. Another interpretation could be that in early stages of golf, the learners are still unable to take advantage of the positive aerodynamic effects of the ball spins and avoid hitting the ground [54]. This type of trajectory seems to be more adopted on the pitch shot, allowing for higher carrying distances to be achieved [55]. The dynamic loft is affected by the attack angle, and so it is likely that the negative attack angle led to a decrease in the dynamic loft [55]. The face angle also revealed differences between training interventions in the 35 m and 50 m distance. Accordingly, a change was found from negative to positive angles after the DL intervention, suggesting that golfers adopted different strategies that may result from exploring different movement patterns under variability [17].

It was expected that golfers adopt different strategies to swing according to the distance [55]. For example, Kim, Youm, Son, Lee, and Kim [55] explored the impact of distance (20 m and 30 m) and golf shot (pitch and lob) and found differences between shots for ball position, vertical force distribution, and club head speed among others. When considering the distances, the club speed has a major role [55]. Interestingly, when comparing the RB group with the DL group, major differences were identified for the 50 m distance. For this distance, differences for the score, carry distance, and club speed in favor of the DL group were shown. Considering the important role of club head speed in achieving higher carry distance [50], it is not surprising that if the DL group was able to improve the club head speed; they were also able to enhance the carry distance [5,7]. These results are in line with those found for speed skating after a DL intervention [56], whereas the advantages of this approach are more evident in more complex scenarios, with increased information flow, such as during shots for long distances. A similar effect is assigned to the duration of intervention [37]. A previous study explored the acute effects of a traditional intervention based on repetitive and corrective instruction and an intervention sustained by additional variability (i.e., emphasizing the movement error); the findings revealed improvements in golfers club speed, even after the retention test [12]. It was suggested that by amplifying the movement error, which corresponds to the increase in observed fluctuations in DL based on stochastic resonance [34], the golfers developed the ability to promote adjustments to achieve the target behavior contributing to better performances [12], thus it may be speculated that the fluctuations imposed by the DL approach promoted similar effects on the golfers from this study. In contrast, more repetitive approaches are primarily based on information about the negativity of movement errors compared to the desired target. This prevents the golfer from exploring the edges of the neural solution space by making non-representative movements, thus using the interpolation ability of neural networks in the long run to react robustly against future internal and external perturbations [12,34].

### 4.2. Effects of DL Practicing between Low and High Handicap Golfers

Earlier research showed that football players with different experience levels, showed different adaptions as a result of being exposed to DL interventions [30,35]. In this respect, it may also be expected that golfers with distinct handicap levels would also reveal differences in their development following a DL intervention. In general, the results between the low and high handicap players belonging to the DL group revealed higher effects when considering the further distances (see Table 4). Accordingly, effects in score and attack angle were found for all distances, while differences in club speed, face angle, and dynamic loft were found for the 35 and 50 m distance. In general, the high handicap group improved the score points more so in all conditions. These results corroborated earlier reports that showed less expert soccer players seem to amplify their learning following interventions grounded in variability, possibly as a result of less time spent on structured practice [30,35]. Therefore, it may be possible that the fluctuations presented for the players have emphasized the high handicap group movement exploration [26,28,33], allowing the players’ movement system to understand how to better address the movement towards the desired goal. Despite these improvements, the results also showed improvements in the low handicap group club speed for the 50 m distance. While both low and high handicap golfers seem to adapt the biomechanics of their movements to increase the club heads’ maximum acceleration, maximum speed, and amplitude of the downswing phase, it seems that low handicap players have a better understanding of the association between impact force production and the club kinematic patterns [2]. Thus, it seems that the low handicap group adjusted the angle-related variables (i.e., attack angle, face angle, and dynamic loft) to increase the club speed. For instance, the low handicap group following the DL intervention increased the negative attack angle (i.e., decrease in ball height in relation to the ground) and, consequently, decreased the dynamic loft as both variables are related [55]. Altogether, these variables accounted for the increase in the club speed. Considering that players were exposed to a wide range of movement fluctuations, the corresponding effects may result from the higher ability of low handicap players in evaluating the distance and controlling the club head impact, such as the angle variables, when compared to players with higher handicap level [2,3,54].

### 4.3. Effects of RB Approach between Low and High Handicap Golfers

From shorter distances, the low handicap group seems to benefit from practicing under more repetitive approaches, as they both improved the score and club speed for the 20 and 35 m distance, while they also improved the carry distance for the latest. In contrast, the high handicap group revealed better results for the score and club speed for the 50 m distance. One major difficulty in golf is hitting the ball towards the pretended direction and distance [2,3]. For the players to achieve a lower handicap or higher score, it requires the ability to achieve both the target distance and direction. Previous reports have already suggested that low handicap golfers are better in controlling the club head kinematics, allowing them to apply a higher power when hitting the ball [2]. In contrast, high handicap players are likely to fail to use the rotational power from the trunk muscles, leading towards lower efficient swing kinematics, and consequently, lower club speed [57]. That is, while there could be no differences identified in terms of time to contact and time to peak velocity between groups, in turn, the time spent on each swing phase is different between groups, contributing to a higher peak velocity at the moment of impact by the low handicap group [8]. This higher efficiency allows the ball to roll compared to sliding, leading towards less energy loss [8]. Complementarily, low handicap golfers display different visual search strategies, as they explore fewer fixation numbers but those of higher duration compared to their lower level counterparts [9,53]. For example, low handicap players are able to decrease the number of fixation numbers and use specific environmental cues (e.g., being aware of the contours and how it will affect the ball roll, using the tree to estimate distances), while high handicap players are more focused on the shot action, that is, how to perform the swing and properly adjust the body movement [58]. These differences may help to explain the results found in this study, mainly for the 20 m and 35 m distance. However, distinct results were identified for the 50 m distance, as the improvements were mostly evident for the high handicap group. Firstly, it may be possibly that this visual search behavior strategy may be weak when considering further distances; however, further research is required to deeper explore this issue. At this point, it also may be important to consider that high handicap players may engage in training sessions focused on lower distances to decrease movement errors during the earlier stages of learning. For example, Maxwell et al. [59] founded that golfers revealed better performances in the golf putt shot when progressing from short to long distances by adopting an errorless approach, as decreasing the errors during an early stage of learning seem to emphasize the implicit learning, contributing to better results when performing. Thus, it may be possible that this process contributed to the higher rate of improvement for long-distances in high handicap players.

## 5. Study Constraints as Inspiration for Further Studies

Whilst this study aimed to reveal important practical applications to support golf coaches’ practices, due to the character of the study and the chosen statistics, no claim for generalization can be made. Consequently, the study has constraints that are worth considering in follow-up studies. For example, previous reports have explored the kinetic movement patterns of both low and high handicap golfers [3], which would provide additional detail on how they adapted their movement behavior as a result of the intervention grounded in variability, and thus it should be included in further studies within this domain. Another question would be to study whether the velocity improvement was based on taking advantage of stretch shortening cycles or whether a more precise downswing or different coordination patterns occurred. The question results on whether the effects are more muscular or more neuronal. Additionally, one main advantage of the DL approach is the higher retention when compared to more repetitive approaches. In this respect, further studies should not only include a retention test, but also include intermediate measures to understand the temporal effect of DL in the pitching performance. Likewise, coaches and practitioners would also benefit from further studies exploring the effects of DL in other golf skills (e.g., golf put), distances (e.g., <10 m, >100 m) or players with different characteristics (e.g., age, sex, level, height, weight). Accordingly, in the present study, the players from the DL and RB groups have shown similar profile in most characteristics. However, differences were found for the starting age of golf practice. Considering that previous sports experiences play an important role in players’ performance and their ability to adapt to variability-based interventions [60], future studies may also include the players’ backgrounds as a study scope.

## 6. Conclusions

Overall, the findings from this study suggested that the intervention grounded in real self-organized variability, where no explicit information about the solution is provided, was effective in improving golfers’ performance, for low and high handicap golfers. While there were higher improvement rates for the DL group in all distances relative to the constrained repetitive group, this was especially evident when considering the longest distances. Coaches can therefore further explore the DL approach to improve player performance by relying not only on improved swing technique, but also on improved adaptability, which becomes more evident as distance increases. When considering the effect of variability according to the level of performance, coaches may use DL to improve high handicap score performances, while using it with experts to enhance movement adaptability as expressed by the changes in the attack and face angle, as well as the dynamic loft. Whether the improvement rates become even higher once they are adapted specifically to the performance level or individual [27,34] will be revealed in future studies. Here, additional support is provided for the advantageous effect of including movements into the learning process that are classically considered to be erroneous or not representative. The effects of the more constrained, repetitive, and guided approach also retrieved improvements when considering just the comparisons from pre- to post-test measurements but to a smaller extent. More specifically, low handicap players seem to benefit from this approach in lower distances (i.e., 20 m and 35 m), while high handicap players benefit at higher distances (i.e., 50 m). Whether a shift in the fluctuations towards velocity and rhythm parameters for the low handicap golfers would lead to comparable progress is a possibility to be tested. While coaches may adjust the adequate approach according to the individual golfer’s profile, in general, the results from this study once more suggest that DL can be an effective strategy to enhance even advanced golfers’ performance.

## Figures and Tables

**Table 1:** Golfers’ characteristics according to the different training groups.

Golfers Characterization	DL Group		RB Group	
	Low Handicap (*n* = 7)	High Handicap (*n* = 8)	Low Handicap (*n* = 5)	High Handicap (*n* = 9)
Age (years)	17.71 ± 1.67	14.00 ± 1.32	17.40 ± 1.62	15.78 ± 2.74
Body mass (kg)	73.43 ± 6.34	56.0 ± 8.47	63.60 ± 6.53	66.0 ± 8.11
Height (cm)	177.14 ± 8.74	167.50 ± 7.98	172.4 ± 6.09	172.0 ± 6.16
Handicap	0.50 ± 1.57	20.05 ± 10.69	2.06 ± 1.22	17.39 ± 10.84
Playing experience (years)	10.57 ± 1.84	4.38 ± 2.06	9.0 ± 2.10	6.67 ± 3.30
Starting age practicing golf	5.00 ± 2.00 *	6.50 ± 2.69	2.80 ± 1.60	5.22 ± 2.82

Note: * represent statistically significant effects between the DL and the RB group (*p* ≤ 0.05).

**Table 2:** Methodical differences between the control and DL group.

Session Number	DL Group	RB Group
1	Short grass, the player had to collect the ball in different places from the shot location (i.e., considering a 2 m distance, the player needed to pick the ball in the back, the right, the left). Amount of practice = 27 executions.	Short Grass, 9 shots for each distance (*n* = 9 for 20 m; following by *n* = 9 for 35 m; and ending with *n* = 9 m for the 50 m; in sum = 27 reps per session).
2	Short grass, varying the distance (i.e., 23 m, 33 m, 47 m). Amount of practice = 27 executions.	
3	Varying the surface (i.e., sand). Amount of practice = 27 reps.	
4	Varying the surface (i.e., high grass). Amount of practice = 27 executions.	
5	Varying the iron (i.e., lower loft). Amount of practice = 27 executions.	
6	Varying the ball position according to the feet (front, back, side). Amount of practice = 27 executions.	
7	Varying the iron (i.e., lower size). Amount of practice = 27 executions.	
8	Varying the type of ball (i.e., tennis ball and beach tennis). Amount of practice = 27 executions.	
9	Performing with visual restriction (i.e., using an eye band covering the dominant eye). Amount of practice = 27 executions.	
10	Varying the surface (i.e., sand, short and high grass). Amount of practice = 27 executions.	

**Table 3:** Descriptive (M ± SD; Raw; ±95% CI) and inferential results for the golf shot performance according to the DL and RB group.

Variables	RB Group		DL Group		RB vs. DL Group Difference of Means	*p*
	Pre-Test	Post-Test	Pre-Test	Post-Test		
	(M ± SD)	(M ± SD)	(M ± SD)	(M ± SD)	(Raw; ±95% CI)	
20 m Distance						
Score (points)	53.25 ± 34.79	67.42 ± 24.95	57.36 ± 30.01	72.53 ± 25.66	1.00 ± 8.33	0.114
Club Speed (m·s^−1^)	33.06 ± 3.16	32.51 ± 2.70	32.96 ± 3.49	32.47 ± 3.05	0.34 ± 1.05	0.824
Carry Distance (m)	16.63 ± 5.04	17.82 ± 3.32	16.68 ± 4.50	18.03 ± 3.25	0.15 ± 1.25	0.601
Side (m)	−0.21 ± 0.76	−0.25 ± 0.80	0.04 ± 0.98	0.07 ± 1.13	0.08 ± 0.25	**0.031**
Attack Angle (°)	−4.61 ± 2.62	−4.01 ± 1.95	−3.86 ± 1.79	−4.78 ± 2.03	−1.18 ± 0.66	**<0.001**
Face Angle (°)	−0.89 ± 3.05	−1.16 ± 3.27	−1.19 ± 3.46	−0.27 ± 3.33	1.06 ± 1.12	0.085
Dynamic Loft (°)	43.23 ± 8.56	45.06 ± 8.62	45.71 ± 5.58	44.94 ± 5.91	−1.13 ± 2.81	0.588
35 m Distance						
Score (points)	52.50 ± 34.12	64.54 ± 29.47	56.98 ± 31.54	63.57 ± 25.24	−5.44 ± 9.44	0.677
Club Speed (m·s^−1^)	46.08 ± 3.90	45.29 ± 4.32	46.08 ± 3.82	45.74 ± 3.57	0.14 ± 1.30	0.467
Carry Distance (m)	29.93 ± 7.47	31.34 ± 6.05	30.51 ± 6.67	32.10 ± 5.18	0.17 ± 2.18	0.227
Side (m)	0.17 ± 1.33	0.15 ± 1.28	0.27 ± 2.00	0.46 ± 1.69	0.22 ± 0.49	0.089
Attack Angle (°)	−4.22 ± 2.64	−3.73 ± 1.91	−3.28 ± 2.26	−4.42 ± 2.00	−1.57 ± 1.01	**0.033**
Face Angle (°)	−0.06 ± 3.21	0.41 ± 2.70	−0.53 ± 3.06	3.43 ± 11.33	4.13 ± 2.45	**0.004**
Dynamic Loft (°)	42.77 ± 7.35	45.09 ± 6.48	43.74 ± 6.17	41.24 ± 12.63	−6.48 ± 3.35	**<0.001**
50 m Distance						
Score (points)	47.56 ± 32.99	56.58 ± 30.23	48.99 ± 34.73	68.16 ± 25.97	10.15 ± 9.5	**<0.001**
Club Speed (m·s^−1^)	57.79 ± 4.18	56.32 ± 3.38	56.46 ± 4.39	57.47 ± 3.97	2.24 ± 1.21	**0.003**
Carry Distance (m)	44.83 ± 8.52	45.45 ± 8.07	43.55 ± 9.90	47.95 ± 6.12	3.78 ± 2.57	**0.002**
Side (m)	0.54 ± 3.92	0.30 ± 2.19	0.12 ± 2.34	0.30 ± 1.69	0.43 ± 0.91	0.946
Attack Angle (°)	−4.06 ± 2.64	−3.61 ± 2.32	−3.51 ± 2.43	−4.78 ± 2.21	−1.98 ± 0.86	**<0.001**
Face Angle (°)	0.68 ± 3.29	0.32 ± 3.43	−0.22 ± 3.24	3.30 ± 11.59	4.41 ± 2.46	**0.003**
Dynamic Loft (°)	42.31 ± 8.08	44.86 ± 7.35	43.88 ± 6.58	41.06 ± 12.38	−6.27 ± 3.21	**<0.001**

Note: DL = differential learning; RB = repetitive-based group; M = mean; SD = standard deviation; CI = confidence interval. Bold values represent statistically significant effects (*p* ≤ 0.05).

**Table 4:** Descriptive (M ± SD; Raw; ±95% CI) and inferential results for the golf shot performance differentiated by the low and high handicap group within the DL group.

Variables	Low Handicap Group		High Handicap Group		Low vs. High Handicap Difference in Means	*p*	Cohen’s d [95% CI]
	Pre-Test	Post-Test	Pre-Test	Post-Test			
	(M ± SD)	(M ± SD)	(M ± SD)	(M ± SD)	(Raw; ±95% CI)		
20 m Distance							
Score (points)	45.43 ± 31.56	63.14 ± 29.11	71.50 ± 20.64	83.27 ± 15.23	−5.94 ± 9.47	**0.002**	−0.21 [−0.55; 0.13]
Club Speed (m·s^−1^)	33.92 ± 4.29	32.27 ± 3.59	32.11 ± 2.30	32.69 ± 2.31	2.02 ± 1.51	0.28	0.61 [0.16; 1.07]
Carry Distance (m)	15.93 ± 5.74	17.41 ± 4.04	17.54 ± 2.16	18.73 ± 1.76	−0.28 ± 1.39	0.066	−0.07 [−0.42; 0.28]
Side (m)	0.10 ± 1.28	0.10 ± 1.50	−0.02 ± 0.42	0.05 ± 0.44	0.07 ± 0.38	0.993	0.07 [−0.28; 0.42]
Attack Angle (°)	−3.57 ± 2.15	−4.11 ± 2.31	−4.08 ± 1.46	−5.46 ± 1.41	−0.26 ± 0.86	**0.035**	−0.14 [−0.58; 0.31]
Face Angle (°)	−0.64 ± 4.76	−0.55 ± 4.28	−1.61 ± 1.88	0.01 ± 2.01	1.46 ± 1.63	0.086	0.43 [−0.05; 0.91]
Dynamic Loft (°)	43.87 ± 6.57	43.84 ± 7.56	47.11 ± 4.22	46.02 ± 3.36	−2.04 ± 2.77	0.653	−0.35 [−0.83; 0.13]
35 m Distance							
Score (points)	48.28 ± 34.57	57.45 ± 28.01	66.93 ± 24.34	70.57 ± 19.61	−5.53 ± 11.61	**0.007**	−0.19 [−0.60; 0.21]
Club Speed (m·s^−1^)	46.55 ± 4.59	45.26 ± 4.41	45.60 ± 2.79	46.27 ± 2.25	2.26 ± 1.62	**0.035**	0.61 [0.17; 1.04]
Carry Distance (m)	29.83 ± 8.56	31.58 ± 6.20	31.30 ± 3.31	32.7 ± 3.64	−0.34 ± 2.79	0.126	−0.06 [−0.52; 0.41]
Side (m)	0.36 ± 2.62	0.57 ± 2.11	0.16 ± 0.84	0.34 ± 1.02	−0.03 ± 0.72	0.466	−0.02 [−0.40; 0.37]
Attack Angle (°)	−3.66 ± 2.75	−3.85 ± 1.97	−2.97 ± 1.73	−5.14 ± 1.81	−1.58 ± 1.47	**0.045**	−0.73 [−1.42; −0.05]
Face Angle (°)	−0.18 ± 3.95	0.31 ± 3.00	−0.86 ± 1.84	6.74 ± 15.33	7.78 ± 4.28	**0.001**	0.93 [0.42; 1.45]
Dynamic Loft (°)	42.16 ± 7.95	44.14 ± 5.35	45.24 ± 3.16	38.17 ± 16.78	−9.64 ± 5.09	**0.005**	−0.96 [−1.47; −0.45]
50 m Distance							
Score (points)	35.55 ± 34.07	60.54 ± 28.52	64.36 ± 28.76	76.87 ± 19.51	−12.47 ± 12.94	**<0.001**	−0.40 [−0.82; 0.02]
Club Speed (m·s^−1^)	55.77 ± 4.99	56.500 ± 3.97	57.02 ± 3.77	58.62 ± 3.69	1.86 ± 1.52	**<0.001**	0.44 [0.08; 0.80]
Carry Distance (m)	40.78 ± 11.96	47.16 ± 7.43	46.72 ± 5.38	48.85 ± 4.01	−4.24 ± 3.31	2.4	−0.51 [−0.91; −0.11]
Side (m)	−0.04 ± 2.79	0.18 ± 1.98	0.29 ± 1.70	0.45 ± 1.28	−0.06 ± 0.97	0.266	−0.03 [−0.50; 0.45]
Attack Angle (°)	−3.03 ± 3.04	−4.12 ± 2.48	−3.83 ± 1.88	−5.52 ± 1.56	−0.65 ± 1.32	**0.05**	−0.28 [−0.84; 0.28]
Face Angle (°)	−0.36 ± 4.30	−0.07 ± 2.69	−0.12 ± 2.05	7.13 ± 15.91	7.08 ± 4.16	**0.002**	0.83 [0.34; 1.31]
Dynamic Loft (°)	42.76 ± 8.86	43.69 ± 6.03	44.77 ± 3.77	38.07 ± 16.48	−7.36 ± 4.81	**0.018**	−0.74 [−1.22; −0.26]

Note: DL = differential learning; M = mean; SD = standard deviation; CI = confidence interval. Bold values represent statistically significance (*p* ≤ 0.05).

**Table 5:** Descriptive (M ± SD; Raw; ±95% CI) and inferential results for the golf shot performance differentiated by the low and high handicap group within the RB group.

Variables	Low Handicap Group		High Handicap Group		Low vs. High Handicap Difference in Means	*p*	Cohen’s d [95% CI]
	Pre-Test	Post-Test	Pre-Test	Post-Test			
	(M ± SD)	(M ± SD)	(M ± SD)	(M ± SD)	(Raw; ±95% CI)		
20 m Distance							
Score (points)	53.88 ± 34.03	61.58 ± 27.03	52.12 ± 36.44	77.94 ± 16.23	18.12 ± 13.18	**<0.001**	0.60 [0.16; 1.03]
Club Speed (m·s^−1^)	32.41 ± 3.37	31.70 ± 2.75	34.12 ± 2.47	33.85 ± 2.03	0.65 ± 1.45	**<0.001**	0.22 [−0.27; 0.71]
Carry Distance (m)	23.50 ± 4.68	23.30 ± 3.80	16.85 ± 5.62	18.40 ± 2.04	0.55 ± 2.04	0.12	0.13 [−0.35; 0.61]
Side (m)	−0.23 ± 0.87	−0.27 ± 0.92	−0.15 ± 0.51	−0.23 ± 0.54	−0.04 ± 0.29	0.896	−0.05 [−0.43; 0.32]
Attack Angle (°)	−4.23 ± 2.42	−3.66 ± 1.86	−5.11 ± 2.83	−4.57 ± 2.00	0.02 ± 1.08	0.144	0.01 [−0.45; 0.47]
Face Angle (°)	−0.69 ± 3.45	−1.39 ± 3.85	−1.18 ± 2.36	−0.77 ± 1.93	1.12 ± 1.62	0.39	0.35 [−0.16; 0.86]
Dynamic Loft (°)	43.30 ± 8.19	45.00 ± 9.33	43.15 ± 9.17	45.18 ± 7.39	1.33 ± 5.29	0.774	0.15 [−0.46; 0.77]
35 m Distance							
Score (points)	48.29 ± 36.70	57.2 ± 31.59	60.08 ± 27.64	77.74 ± 19.33	8.75 ± 14.5	**<0.001**	0.27 [−0.18; 0.73]
Club Speed (m·s^−1^)	45.81 ± 4.41	44.42 ± 4.97	46.52 ± 2.84	46.87 ± 2.01	1.61 ± 1.80	**0.002**	0.39 [−0.05; 0.82]
Carry Distance (m)	36.6 ± 8.44	25.80 ± 6.90	30.37 ± 5.41	33.26 ± 3.46	2.17 ± 2.99	**0.005**	0.32 [−0.12; 0.75]
Side (m)	0.38 ± 1.46	0.08 ± 1.47	−0.20 ± 0.96	0.28 ± 0.83	0.78 ± 0.57	0.422	0.60 [0.16; 1.03]
Attack Angle (°)	−3.54 ± 2.58	−3.31 ± 2.08	−5.25 ± 2.42	−4.34 ± 1.44	0.87 ± 1.40	0.222	0.37 [−0.23; 0.97]
Face Angle (°)	0.51 ± 3.62	0.29 ± 3.18	−0.94 ± 2.24	0.60 ± 1.66	1.58 ± 1.53	0.49	0.53 [0.02; 1.04]
Dynamic Loft (°)	42.14 ± 8.43	45.37 ± 7.02	43.72 ± 5.28	44.62 ± 5.53	−3.93 ± 3.62	0.289	−0.56 [−1.08; −0.04]
50 m Distance							
Score (points)	43.03 ± 33.01	52.33 ± 29.95	55.70 ± 31.68	64.22 ± 29.49	−0.78 ± 15.6	**0.05**	−0.02 [−0.51; 0.47]
Club Speed (m·s^−1^)	57.21 ± 4.37	55.83 ± 3.83	58.68 ± 3.74	57.08 ± 2.35	−0.15 ± 1.85	**0.012**	−0.04 [−0.52; 0.45]
Carry Distance (m)	42.00 ± 9.41	42.20 ± 8.59	45.20 ± 6.84	46.64 ± 7.08	1.29 ± 3.86	0.204	0.15 [−0.31; 0.61]
Side (m)	1.07 ± 3.10	0.31 ± 2.55	−0.40 ± 4.96	0.28 ± 1.33	1.44 ± 1.73	0.825	0.45 [−0.09; 0.99]
Attack Angle (°)	−3.57 ± 2.96	−3.20 ± 2.50	−4.91 ± 1.71	−4.19 ± 1.91	0.12 ± 1.28	0.118	0.05 [−0.46; 0.56]
Face Angle (°)	1.38 ± 3.64	0.58 ± 4.06	−0.36 ± 2.36	−0.10 ± 2.06	0.99 ± 1.44	0.191	0.29 [−0.13; 0.72]
Dynamic Loft (°)	40.91 ± 9.88	44.65 ± 9.11	44.38 ± 3.32	45.18 ± 3.14	−2.90 ± 3.34	0.77	−0.37 [−0.80; 0.06]

Note: RB = repetitive-based group; M = mean; SD = standard deviation; CI = confidence interval. Bold values represent statistically significant effects (*p* ≤ 0.05).

## Data Availability

In order to protect the subjects’ confidentiality and privacy, data are only available on request. Interested researchers may contact the board from the Research Center in Sports Sciences, Health Sciences and Human Development to request access to the data (cidesd.geral@utad.pt).
